# Real-world efficacy and safety profile of low-dose cadonilimab in recurrent or metastatic cervical cancer as first-line therapy

**DOI:** 10.3389/fimmu.2026.1871890

**Published:** 2026-08-18

**Authors:** Xiaoshi Liu, Jing Zheng, Can Zhang, Xunwei Shi, Liping Peng, Dengfeng Wang, Guonan Zhang

**Affiliations:** 1 Department of Gynecologic Oncology, Sichuan Clinical Research Center for Cancer, Sichuan Cancer Hospital & Institute, Sichuan Cancer Center, University of Electronic Science and Technology of China, Chengdu, China; 2 Department of Oncology, The Affiliated Hospital, Southwest Medical University, Luzhou, China

**Keywords:** first-line therapy, low-dose cadonilimab, real world, recurrent or metastatic cervical cancer, resource-constrained settings

## Abstract

**Background:**

Cadonilimab, a bispecific PD-1/CTLA-4 antibody, has activity in recurrent/metastatic cervical cancer (R/M CC), but high costs limit accessibility, especially in low- and middle-income countries. Hence, we aimed to assess whether lower dose of cadonilimab may achieve comparable clinical outcomes with improved tolerability.

**Methods:**

This retrospective real-world study evaluated 137 R/M CC patients treated with cadonilimab 6mg/kg every three weeks as first-line therapy at Sichuan Cancer center from January 1, 2023, to September 30, 2025. The primary endpoint was the objective response rate (ORR). Secondary endpoints included the disease control rate (DCR), progression-free survival (PFS), overall survival (OS), and safety.

**Results:**

Among 133 efficacy-evaluable patients, the ORR was 55.6%, consisting of complete and partial responses in 10.5% and 45.1% of patients, respectively. The DCR was 83.5%. In patients aged ≥65 years, the ORR and DCR were 47.8% and 87.0%, respectively. The 12-month OS rate was 92.1% (95% CI, 86.5%-98.0%), and the 12-month PFS rate was 67.3% (95% CI, 58.4%-77.4%). Grade ≥3 treatment-related adverse events occurred in 78.8% and the most common were neutropenia, anemia, and lymphopenia. While immune-related adverse events were observed in 35.0% of patients, severe events were rare, occurring in only 3.6%. Furthermore, clinical outcomes and safety profiles were comparable across different chemotherapy regimens.

**Conclusions:**

Low-dose cadonilimab demonstrates robust efficacy and manageable safety in R/M CC, with encouraging outcomes in elderly patients. Prospective randomized trials are needed to validate the efficacy of dose-reduced strategies.

## Highlights

- In real-world R/M cc, low-dose cadonilimab delivers robust antitumor activity consistent with phase III data.
- Low-dose cadonilimab shows controllable toxicity and rare high-grade iRAEs.
- Low-dose cadonilimab may improve treatment accessibility in resource-limited settings.

## Background

Cervical cancer (CC) represents the fourth most common cancer among women globally (1). Despite being largely preventable through human papillomavirus (HPV) vaccination and screening programs, cervical cancer disproportionately affects low- and middle-income countries (LMICs), where 88% of cases and 94% of deaths occur (2, 3). This disparity reflects limited access to prevention, early detection, and treatment resources. Therefore, in resource-limited settings lacking routine screening infrastructure, over 70% of cervical cancer cases present at advanced or metastatic stages (4, 5). Conversely, in developed nations, approximately 5% of cervical cancer patients are diagnosed with metastatic disease. Furthermore, despite standard treatment with concurrent chemoradiation followed by brachytherapy for locally advanced disease, recurrence occurs in roughly one-third of patients. Consequently, the vast majority of recurrent or metastatic cervical cancer (R/M CC) cases that are ineligible for locoregional salvage therapies are deemed incurable, carrying a dismal prognosis.

The integration of immune checkpoint inhibitors has improved outcomes in high-income settings. The phase III KEYNOTE-826 trial demonstrated that pembrolizumab plus chemotherapy ± bevacizumab significantly prolonged OS in patients with PD-L1–positive R/M CC, leading to its adoption as a new global standard (6). However, this advance has not translated into equitable benefit. Full-dose anti–PD-1 monotherapy typically costs more than $150,000 annually per patient in the United States—a figure exceeding the per capita gross national income of most LMICs by orders of magnitude (7). Cadonilimab, a tetravalent bispecific antibody targeting both PD-1 and CTLA-4, has demonstrated its therapeutic potential in small-sample, early-phase clinical trials, which was ultimately confirmed in the pivotal phase III trial (COMPASSION-16) (8, 9). However, the gap between randomized controlled trial efficacy and real-world clinical application remains substantial, particularly for novel immunotherapies in resource-limited settings, where strict eligibility criteria exclude patients with comorbidities, advanced age, or socioeconomic barriers. Given the urgent need to improve global equity in cancer care, evaluating whether reduced-dose cadonilimab—such as 6 mg/kg administered every 3 weeks—can maintain clinical benefit while enhancing affordability is both scientifically plausible and ethically imperative. Real-world evidence is critical to inform dose-optimization strategies that preserve efficacy without compromising safety.

## Materials and methods

### Study design and participants

This non-randomized retrospective study evaluated the antitumor activity and safety of cadonilimab in patients with R/M CC across Sichuan cancer center between January 1, 2023 and September 30, 2025. Inclusion criteria were as follows: (1) histologically confirmed R/M CC with no prior systemic therapy for persistent, recurrent, or metastatic disease; (2) at least one measurable tumor lesion according to the Response Evaluation Criteria in Solid Tumors (RECIST) version 1.1; and (3) receipt of at least one cycle of cadonilimab treatment (6 mg/kg Q3W). Key exclusion criteria included a history of other malignancies, concurrent malignancies, or incomplete clinical data. All patients were managed according to routine clinical practice and patient preference. Follow-up was conducted for at least six months after treatment initiation, unless death occurred earlier. Notably, at the time of patient enrollment, first-line cadonilimab was not yet reimbursed by China’s National Reimbursement Drug List for the majority of the enrollment period, and the standard 10 mg/kg regimen imposed prohibitive out-of-pocket costs for most patients. Consequently, a standard-dose comparator was not feasible in this real-world setting, and our study should be viewed as an exploratory analysis rather than a comparative effectiveness trial.

### Procedures

Detailed baseline characteristics, including demographic information, disease history, and prior treatment records, were collected from electronic medical records and verified through direct interviews with physicians and patients. Treatment-related data, including chemotherapy regimens, cadonilimab dosage, dosage adjustments, evaluation intervals, and reasons for treatment discontinuation, were systematically extracted from hospital information systems. Adverse events (AEs) were collected from the date of cadonilimab initiation until the end of the study.

### Outcome measures

Tumor responses were assessed by gynecologic oncologists and radiologists according to the Response Evaluation Criteria in Solid Tumors (RECIST) version 1.1 (10, 11). The primary endpoints were objective response rate (ORR), defined as the proportion of patients with measurable disease achieving complete response (CR) or partial response (PR), and disease control rate (DCR), defined as the proportion of patients achieving CR, PR, or stable disease (SD). Secondary endpoints included progression-free survival (PFS), measured from the date of initial cadonilimab administration to disease progression (PD) or death from any cause, whichever occurred first, and overall survival (OS), defined as the time from treatment initiation to death from any cause. Adverse events (AEs) and immune-related adverse events (irAEs) were recorded and graded in accordance with the National Cancer Institute Common Terminology Criteria for Adverse Events (NCI-CTCAE) version 5.0 (12). Additionally, exploratory analyses were conducted to evaluate pooled effectiveness and safety in specific patient subgroups underrepresented in pivotal clinical trials, including patients aged ≥65 years and those receiving different concomitant chemotherapy regimens.

### Statistical analyses

Continuous variables are presented as median with range and interquartile range. Categorical variables are summarized using absolute numbers and corresponding percentages. Time-to-event endpoints, including PFS and OS, were estimated by the Kaplan–Meier method, with 95% confidence intervals derived using the Brookmeyer–Crowley approach. Binary efficacy outcomes, namely objective response rate and disease control rate, were reported as proportions, and their 95% confidence intervals were calculated via the Clopper–Pearson exact method. For comparisons between paclitaxel-based and gemcitabine-based chemotherapy regimens, we performed 1:3 nearest-neighbor propensity score matching using a caliper of 0.2 times the standard deviation of the propensity score. The propensity score was estimated using a logistic regression model that included the following baseline covariates: age, Eastern Cooperative Oncology Group performance status (ECOG PS), disease status at study entry, prior chemoradiotherapy, bevacizumab use during the study. Standardized mean differences (SMD) were calculated to assess covariate balance, with an SMD of less than 0.1 considered indicative of adequate balance. Log‐rank test examined the difference between two survival curves and Cloglog transformation test was performed to compare survival at a fixed time point. R version 4.3.2 was used for data analysis.

## Results

### Patients and treatment characteristics

Between January 1, 2023, and September 30, 2025, 137 women with R/M CC were screened, all 137 patients were included in the safety analysis, and 133 patients who underwent at least one post-baseline tumor assessment were included in the effectiveness analysis. Overall, the median age was 54 years (IQR 47–61), and 17.5% were aged ≥ 65 years. 82 (65.9%) participants had an Eastern Cooperative Oncology Group performance score (ECOG PS) of 1, 107 (78.1%) had squamous cell carcinoma and 105 (76.6%) had metastatic disease at baseline. 68.6% received previous chemoradiotherapy with or without surgery, and 20.4% had previously untreated metastatic disease at study entry. Among the 52 patients who underwent PD-L1 status testing, 11 participants had a PD-L1 CPS lower than 1, 18 had a PD-L1 CPS of 1 or higher, and 23 had a PD-L1 CPS of 10 or higher.

The most frequently administered concurrent chemotherapy regimens comprised paclitaxel in combination with either carboplatin (n = 76, 55.5%) or cisplatin (n = 30, 21.9%). 31 (22.6%) patients received platinum plus gemcitabine regimen. Bevacizumab was used by 96 (70.1%) participants. Radiotherapy was delivered to 42 patients (30.7%), and 12 patients (8.8%) continued cadonilimab beyond disease progression. The baseline characteristics of the patients are listed in **Table 1**.

**Table 1:** Demographic and disease characteristics of the patients at baseline.

Characteristics	All (N = 137)
Age	
Median, years	54.00 (47.00, 61.00)
≥65 years - no. (%)	24 (17.5%)
ECOG performance status - no. (%)	
0	48 (35.0%)
1	82 (59.9%)
2	7 (5.1%)
FIGO stage at initial diagnosis - no. (%)	
I	22 (16.1%)
IIA	9 (6.6%)
IIB	13 (9.5%)
IIIA	4 (2.9%)
IIIB	18 (13.1%)
IIIC	47 (34.3%)
IVA	3 (2.2%)
IVB	21 (15.3%)
Metastatic disease at study entry - no. (%)	
Yes	105 (76.6%)
No	32 (23.4%)
Pathological diagnosis - no. (%)	
Squamous cell carcinoma	107 (78.1%)
Adenocarcinoma	24 (17.5%)
Adenosquamous carcinoma	6 (4.4%)
PD-L1 CPS - no. (%)	
<1	11 (8.0%)
1 to <10	18 (13.2%)
≥10	23 (16.8%)
Unknown	85 (62.0%)
Previous therapy - no. (%)	
Chemoradiotherapy and surgery	27 (19.7%)
Radiotherapy and surgery	4 (2.9%)
Chemoradiotherapy only	67 (48.9%)
Radiotherapy only	5 (3.6%)
Surgery only	6 (4.4%)
None	28 (20.4%)
Bevacizumab use during the study - no. (%)	
Yes	96 (70.1%)
No	41 (29.9%)
Radiotherapy during the study - no. (%)	
Yes	42 (30.7%)
No	95 (69.3%)
Concurrent chemotherapy regimen - no. (%)	
Paclitaxel plus platinum	106 (77.4%)
Gemcitabine plus platinum	31 (22.6%)

### Effectiveness

The median follow-up was 11 (IQR, 9 to 13) months. A total of 48 (35.4%) patients experienced disease progression or had died without disease progression. Among the 137 enrolled patients, 133 were evaluable for efficacy assessment. The best overall response comprised 14 complete responses (CR; 10.5%), 60 partial responses (PR; 45.1%), 37 cases of stable disease (SD; 27.8%), and 21 progressive disease (PD; 15.8%). The objective response rate (ORR; CR+PR) was 55.6% (74/133), and the disease control rate (DCR; CR+PR+SD) was 83.5% (111/133). Among the patients aged 65 years or older, the ORR was 47.8% (1 CR and 10 PR), and the DCR was 87.0%. At the cutoff date, the estimated 12-month OS rates were 92.1% (95% CI, 86.5%-98.0%) and the estimated 12-month PFS were 67.3% (95% CI, 58.4%-77.4%).

### Safety

All 137 participants (100%) experienced treatment-emergent adverse events (TEAEs), with no unexpected adverse events identified. The most frequent grade ≥3 TEAEs were neutropenia [24 (17.5%)], anemia [16 (11.7%)], and lymphopenia [21 (15.3%)]. Immune-related adverse events (irAEs) occurred in 48 participants (35.0%), with hypothyroidism [17 (12.4%)], infusion-related reactions [8 (5.8%)], and myocarditis [7 (5.1%)] being most common. Grade ≥3 irAEs were reported in 5 participants (3.6%), comprising two cases of myocarditis, two of hypocortisolism, and one of pneumonitis. All grade ≥3 irAEs were effectively managed with standard therapeutic interventions. Cadonilimab was permanently discontinued due to irAEs in 15 participants (10.9%). A summary of all AEs is presented in **Table 2**.

**Table 2:** Summary of adverse events in the safety analysis set.

Adverse events	Any grade	Grade 3–5
TRAE occurred in ≥10%		
Anemia	73 (53.3%)	16 (11.7%)
White blood cell count decreased	100 (73.0%)	24 (17.5%)
Neutrophil count decreased	100 (73.0%)	21 (15.3%)
Platelet count decreased	61 (44.5%)	7 (5.1%)
Neuropathy peripheral	53 (38.7%)	4 (2.9%)
Increased hepatic enzymes	49 (35.8%)	5 (3.6%)
Increased bilirubin	37 (27.0%)	4 (2.9%)
Rash	22 (16.1%)	4 (2.9%)
Diarrhea	18 (13.1%)	1 (0.7%)
Nausea	29 (21.2%)	6 (4.4%)
Vomiting	21(15.3%)	3 (2.2%)
Constipation	18 (13.1%)	3 (2.2%)
Decreased appetite	33 (24.1%)	8 (5.8%)
Proteinuria	32 (23.4%)	2 (1.5%)
irAE occurred in ≥2 cases		
Hypothyroidism	17 (12.4%)	2 (1.5%)
Myocarditis	7 (5.1%)	2 (1.5%)
Rash	6 (4.4%)	0
Pneumonitis	5 (3.6%)	1 (0.7%)
Hepatitis	3 (2.2%)	0
Infusion-related reaction	8 (5.8%)	0

### Effectiveness and safety with different chemotherapy regimens

**Figure 1:**
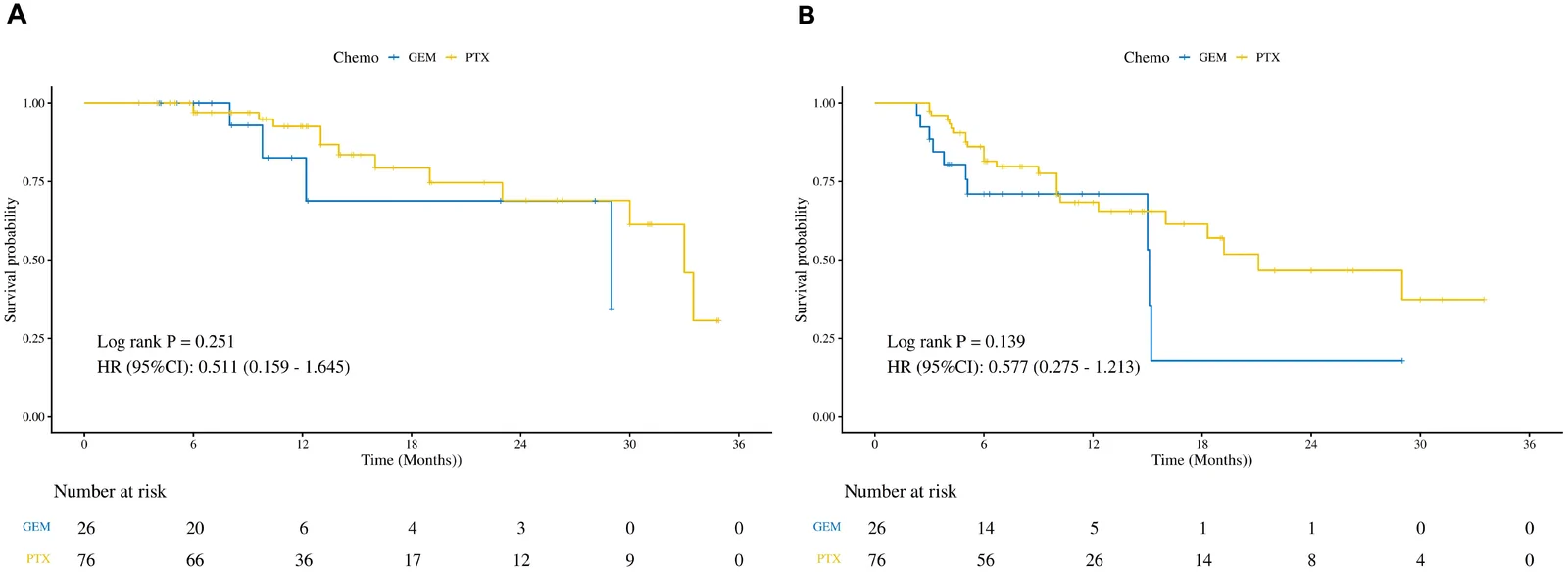
Kaplan–Meier curves of OS **(A)** and PFS **(B)** stratified by different chemotherapy regimens.

As for the different chemotherapy regimens, Nearest propensity-score matching was performed in a 1:3, with a caliper of 0.2. After matching, the final cohort comprised 26 patients receiving gemcitabine-based therapy and 76 patients receiving paclitaxel-based therapy (**Supplementary Table 1**). Cadonilimab combined with the paclitaxel-based regimen resulted in longer PFS (median: 21.1 vs 15.1 months) and OS (median: 33 vs 29 months) when compared with the gemcitabine-based regimen, however, no significant differences were observed between the groups (log-rank p = 0.139 and p = 0.251 for PFS and OS, respectively) (**Figure 1**). The 12-month PFS rates were 68.3% for the paclitaxel group and 71.0% for the gemcitabine group; the corresponding 12-month OS rates were 92.5% and 82.5%. However, no significant differences were observed between the groups (Cloglog p=0.316 and Cloglog p=0.206 for PFS and OS, respectively).

In analysis of adverse events, no significant difference was observed between the 2 treatment groups (**Table 3**). The incidence of grade 3 or higher haematological toxicities in the GEM group was as follows: leukopenia in 11.5%, neutropenia in 11.5%, anemia in 7.7%, and thrombocytopenia in 7.7%. In the PTX group, the corresponding rates were 19.7% for leukopenia, 18.4% for neutropenia, 10.5% for anemia, and 6.6% for thrombocytopenia. Incidence of grade 3 or worse nausea, vomiting, and diarrhea developed in 3.8%, 3.8% and 3.8%, respectively, of the patients in the GEM arm, and in 3.9%, 1.3% and 0%, respectively, of the patients in the PTX arm. Although the difference was not statistically significant, grade 3 or worse gastrointestinal toxicities were more frequent in the GEM arm.

**Table 3:** Adverse events of any cause in either chemotherapy regimen group.

Event	GEM group (N = 26)		PTX group (N = 76)	
	Any grade	Grade 3–5	Any grade	Grade 3–5
Anemia	17 (65.4%)	2 (7.7%)	35 (46.1%)	8 (10.5%)
White blood cell count decreased	18 (69.2%)	3 (11.5%)	55 (72.4%)	15 (19.7%)
Neutrophil count decreased	17 (65.4%)	3 (7.7%)	56 (73.7%)	14 (18.4%)
Platelet count decreased	8 (30.8%)	2 (7.7%)	35 (46.1%)	5 (6.6%)
Neuropathy peripheral	12 (46.2%)	2 (7.7%)	35 (46.1%)	5 (6.6%)
Increased hepatic enzymes	12 (46.2%)	1 (3.8%)	28 (36.8%)	4 (5.3%)
Increased bilirubin	6 (23.1%)	0	17 (22.4%)	2 (2.6%)
Rash	4 (15.4%)	1 (3.8%)	16 (21.1%)	2 (2.6%)
Diarrhea	3 (11.5%)	1 (3.8%)	9 (11.8%)	0
Nausea	6 (23.1%)	1 (3.8%)	14(18.4%)	3 (3.9%)
Vomiting	3 (11.5%)	1 (3.8%)	11 (14.5%)	1 (1.3%)
Constipation	6 (23.1%)	0	6 (7.9%)	3 (3.9%)
Decreased appetite	8 (30.8%)	2 (7.7%)	14 (18.4%)	5 (6.6%)

## Discussion

Cervical cancer remains a stark emblem of global health inequity. Despite being largely preventable through human papillomavirus (HPV) vaccination and screen-and-treat programs, it claims more than 340,000 lives annually—over 90% of which occur in LMICs (2). For patients with recurrent or metastatic disease, the prognosis has historically been poor, with a 5-year survival rate of only approximately 17% (13). While the field of cervical cancer treatment is rapidly evolving: the results from the Keynote-826 and BEATcc trials demonstrate promising efficacy—with median progression-free survival approximately of 12 months and manageable toxicity—access to such cutting-edge therapies remains starkly inequitable (6, 14). The recent approval of cadonilimab, a novel bispecific antibody targeting PD-1 and CTLA-4, marks a significant advance in the immunotherapy of recurrent or metastatic cervical cancer—a disease that disproportionately burdens women in LMICs (9). In this work, we found that strategic exploration of low-dose immune checkpoint inhibition, exemplified by cadonilimab’s unique pharmacokinetic and safety profile, could serve as a pragmatic pathway to expand life-saving treatment access in resource-constrained settings. Nevertheless, we emphasize that these findings are preliminary. The absence of a standard-dose cadonilimab control group—driven by the lack of reimbursement and prohibitive costs during the enrollment period—precludes any direct comparison with the approved regimen. Our data should therefore be interpreted as generating hypotheses for future study, rather than providing definitive evidence of dose equivalence.

Our real-world evidence demonstrates that first-line cadonilimab at a reduced dose of 6 mg/kg achieves a robust objective response rate (ORR) of 55.6% in R/M CC. In the pre-immunotherapy era, the prognosis for patients with R/M CC was extremely poor. Early Gynecologic Oncology Group (GOG) studies reported that single-agent cisplatin, the first established systemic therapy, yielded a median overall survival (OS) of only 6.1 to 7.1 months, with objective response rates ranging from 17% to 38% depending on dose intensity (15, 16). The landmark GOG 169 study (2004) compared cisplatin plus paclitaxel versus cisplatin alone in 260 patients with R/M CC (17). The addition of anti-angiogenic therapy represented the next major advance. GOG 240 showed that adding bevacizumab to chemotherapy significantly improved median OS from 13.3 to 16.8 months (HR 0.77, P = 0.007), leading to FDA approval of bevacizumab in combination with paclitaxel-cisplatin or paclitaxel-topotecan as first-line therapy for R/M CC in 2014 (18). This efficacy signal is not only comparable to historical controls of standard-dose PD-1 inhibitors from landmark phase III trials but also aligns with the pharmacodynamic superiority established in cadonilimab’s own Phase III COMPASSION-16 study. For instance, the KEYNOTE-826 trial reported an ORR of approximately 68.5% in the pembrolizumab arm, albeit in a PD-L1-enriched population (6). Although the ORR observed in our real-world cohort is numerically lower than that reported with the 10 mg/kg dose in the phase III COMPASSION-16 study, this difference is at least partially attributable to key differences in patient composition. Specifically, our study included a considerably higher proportion of adenocarcinoma and other rare or unfavorable pathological subtypes, which are generally associated with poorer treatment responsiveness and less favorable outcomes compared with predominantly squamous cell carcinoma populations enrolled in most phase III trials of immune checkpoint inhibitors.

Pharmacokinetic studies confirm that near-complete PD-1 receptor occupancy on circulating T cells can be achieved at doses substantially below standard regimens. A analysis based on population pharmacokinetic modeling demonstrated no survival difference between nivolumab 240 mg flat-dose and weight-based 3 mg/kg dosing in patients with advanced tumors (19). Chang (20) and Zhao (21) demonstrated comparable clinical outcomes in distinct patient populations: those with non-small cell lung cancer administered pembrolizumab at doses below 2 mg/kg, and those with advanced renal cell carcinoma receiving nivolumab at doses below 2.15 mg/kg. Besides, a recent randomized trial conducted in India (PLANeT) showed similar results (22).

Cadonilimab’s clinical profile is compelling. Phase I dose-escalation data reveal antitumor activity across doses from 1 mg/kg to 10 mg/kg, with no clear dose–response plateau (23). In a pivotal phase II trial involving 111 patients with recurrent or metastatic cervical cancer, cadonilimab 10 mg/kg Q3W as the first-line treatment demonstrated an objective response rate of 79.3%, with a disease control rate of 96.6% (24). Notably, the safety profile was more favorable than that historically observed with combination of anti–PD-1 and anti–CTLA-4: grade ≥3 TRAEs occurred in 73.3% of patients, and only 20.0% of patients experienced grade≥3 irAEs. Immune-related toxicities—such as colitis, hepatitis, and hypophysitis—were markedly reduced. In LMICs, managing grade 3–4 irAEs often requires hospitalization and specialist input—resources absent in 60% of rural clinics (25, 26). In our low-dose cohort, only 3.6% patients experienced grade 3 or higher irAEs, a rate numerically lower than that reported with standard-dose regimens, though cross-trial comparisons are inherently limited by differences in study design and patient populations. Besides, immune-related myocarditis occurred in 5.1% of our real-world cohort, which is an evidently higher rate than pooled registrational safety data of cadonilimab (9). Six of seven myocarditis patients received concurrent bevacizumab, and five had pre-existing hypertension. Our center implemented routine per-cycle cardiac biomarker and ECG testing, which enabled identification of asymptomatic subclinical immune myocarditis that would be overlooked by symptom-triggered monitoring alone (27, 28). Clinicians should strengthen cardiac surveillance for patients treated with cadonilimab combined with bevacizumab or complicated by hypertension.

Our retrospective observational study has inherent methodological constraints that warrant explicit disclosure. First and most importantly, this study lacks a standard-dose cadonilimab comparator group. At the time of patient enrollment, the first-line indication for cadonilimab had not yet been approved by China’s NMPA until May 27, 2025, and no reimbursement was available from the National Reimbursement Drug List in this setting for the study period. The full standard dose (10 mg/kg every 3 weeks) incurred out-of-pocket costs of approximately $3,000–$4,000 per cycle, which was prohibitive for the vast majority of patients in our resource-limited catchment area. As a result, a standard-dose control arm was ethically and practically infeasible. We therefore cannot draw any conclusion regarding non-inferiority or equivalence to the approved 10 mg/kg regimen. Our results should be interpreted as preliminary evidence that, when standard-dose immunotherapy is inaccessible, a reduced-dose approach may still offer clinical benefit over chemotherapy alone. Encouragingly, following the recent NRDL inclusion and NMPA approval of the first-line indication, standard-dose cadonilimab has become increasingly accessible at our institution since January 2026. We are now actively collecting data on patients receiving the standard 10 mg/kg regimen and plan to perform a comparative analysis once the cohort reaches adequate maturity and sample size. This ongoing work will directly address the comparison that the current study cannot make. Second, patient enrollment was influenced by socioeconomic factors, with individuals unable to afford standard-dose cadonilimab receiving the low-dose regimen, potentially introducing systematic bias due to unmeasured confounders such as baseline health-seeking behavior and nutritional status. Third, the relatively modest sample size and single-center design may limit generalizability and statistical power to detect clinically meaningful differences, necessitating cautious interpretation of non-significant findings.

To our knowledge, our study is the first to suggest the effectiveness of low-dose cadonilimab in first-line treatment R/M CC. Further, along with prior studies, this study demonstrated that cadonilimab in combination with various chemotherapy regimens yields comparable clinical efficacy. As the global oncology community strives to implement the WHO’s vision of equitable access to essential cancer medicines, strategies that preserve clinical benefit while reducing cost and toxicity are not merely pragmatic, but imperative. Patients in resource-constrained regions where standard immunotherapy remains out of reach may consider low-dose cadonilimab. Further well-designed prospective studies, such as a non-inferiority phase III trial with sufficient sample size, are needed to confirm the efficacy of low-dose cadonilimab. Our institution is currently enrolling patients receiving standard-dose cadonilimab, and we anticipate that a comparative analysis will be feasible in the next 1–2 years, which we plan to report in a subsequent study.

## Data Availability

The raw data supporting the conclusions of this article will be made available by the authors, without undue reservation.
