# Supplementary material for: Real-world efficacy and safety profile of low-dose cadonilimab in recurrent or metastatic cervical cancer as first-line therapy
Source: Front Immunol. 2026 Aug 18;17:1871890. doi: 10.3389/fimmu.2026.1871890 (PMC13529973; doi:10.3389/fimmu.2026.1871890)
Supplement: Supplementary file 1 [file Table1.docx]

Supplementary Table1 Demographic and disease characteristics of the patients in different chemo regimen group at baseline.

| **Characteristics** | **GEM group** | **PTX group** | **P-value** | **Standardized difference** | |
| --- | --- | --- | --- | --- | --- |
|  | **(N=26)** | **(N=76)** |  | **Before matching** | **After matching** |
| Age (Median, years) | 53.5 (48.0, 63.0) | 53.0 (47.8, 60.3) | 0.65 | 0.24 | 0.10 |
| ECOG (0/1/2) | 10/15/1 | 32/39/5 | 0.93 | 0.04 | 0.02 |
| Prior chemoradiotherapy (Y/N) | 23/3 | 67/9 | 1.00 | 0.02 | 0.01 |
| Metastatic disease at study entry (Y/N) | 20/6 | 61/15 | 0.72 | 0.18 | 0.17 |
| Bevacizumab use during study (Y/N) | 13/13 | 48/28 | 0.26 | 0.13 | 0.06 |
